# Inequality in outcomes for adolescents living with perinatally acquired HIV in sub‐Saharan Africa: a Collaborative Initiative for Paediatric HIV Education and Research (CIPHER) Cohort Collaboration analysis

**DOI:** 10.1002/jia2.25044

**Published:** 2018-02-27

**Authors:** CIPHER Global Cohort Collaboration, Amy L Slogrove, Baylor Botswana, Gabriel Anabwani, Baylor Lesotho, Edith Mohapi, Baylor Malawi, Peter N. Kazembe, Baylor Swaziland, Makhosazana Hlatshwayo, Baylor Tanzania, Mwita Lumumba, Baylor Uganda, Adeodata Kekitiinwa‐Rukyalekere, Christelle Twizere, Marcel Yotebieng, Jean D'amour Sinayobye, Samuel Ayaya, Elizabeth Bukusi, Geoffrey Somi, Rita Lyumuya, Ngonyani Kapella, Mark Urassa, Mark Ssali, Fred Nalugoda, Gary Maartens, Christopher J. Hoffmann, Michael Vinikoor, Eusebio Maceta, Monique van Lettow, Robin Wood, Shobna Sawry, Frank Tanser, Andrew Boulle, Geoffrey Fatti, Sam Phiri, Janet Giddy, Cleophas Chimbetete, Kennedy Malisita, Karl Technau, Brian Eley, Christiane Fritz, Michael Hobbins, Kamelia Kamenova, Matthew P. Fox, François Dabis, Emmanuel Bissagnene, Elise Arrivé, Patrick Coffie, Didier Ekouevi, Antoine Jaquet, Valériane Leroy, Sikiratou Koumakpaï, Marie‐Sylvie N'Gbeche, Kouadio Kouakou, Madeleine Amorissani Folquet, Tanoh François Eboua, Lorna Renner, Fatoumata Dicko, Mariam Sylla, Elom Takassi, Haby Signate‐Sy, Hélène Dior, Diarra Yé, Fla Kouéta, Mohamed Ahmed, Zelalem Habtamu, Kassahun Hailegiorgis, Zenebe Melaku, Mark Hawken, Maureen Kamene Kimenye, Irene N. Mukui, Josue Lima, Antonio Mussa, Américo Rafi Assan, Vincent Mutabazi, Ruben Sahabo, Gisenyi Prison, Gretchen Antelman, Redempta Mbatia, Geoffrey Somi, Matthew Lamb, Denis Nash, Harriet Nuwagaba‐Biribonwoha

**Keywords:** adolescent, HIV, perinatally acquired, sub‐Saharan Africa, Sustainable Development Goals

## Abstract

**Introduction:**

Eighty percent of adolescents living with perinatally and behaviourally acquired HIV live in sub‐Saharan Africa (SSA), a continent with marked economic inequality. As part of our global project describing adolescents living with perinatally acquired HIV (APH), we aimed to assess whether inequality in outcomes exists by country income group (CIG) for APH within SSA.

**Methods:**

Through the CIPHER cohort collaboration, individual retrospective data from 7 networks and 25 countries in SSA were included. APH were included if they entered care at age <10 years (as a proxy for perinatally acquired HIV) and had follow‐up at age >10 years. World Bank CIG classification for median year of first visit was used. Cumulative incidence of mortality, transfer‐out and loss‐to‐follow‐up was calculated by competing risks analysis. Mortality was compared across CIG by Cox proportional hazards models.

**Results:**

A total of 30,296 APH were included; 50.9% were female and 75.7% were resident in low‐income countries (LIC). Median [interquartile range (IQR)] age at antiretroviral therapy (ART) start was 8.1 [6.3; 9.5], 7.8 [6.2; 9.3] and 7.3 [5.2; 8.9] years in LIC, lower‐middle income countries (LMIC) and upper‐middle income countries (UMIC) respectively. Median age at last follow‐up was 12.1 [10.9; 13.8] years, with no difference between CIG. Cumulative incidence (95% CI) for mortality between age 10 and 15 years was lowest in UMIC (1.1% (0.8; 1.4)) compared to LIC (3.5% (3.1; 3.8)) and LMIC (3.9% (2.7; 5.4)). Loss‐to‐follow‐up was highest in UMIC (14.0% (12.9; 15.3)) compared to LIC (13.1% (12.4; 13.8)) and LMIC (8.3% (6.3; 10.6)). Adjusted mortality hazard ratios (95% CI) for APH in LIC and LMIC in reference to UMIC were 2.50 (1.85; 3.37) and 2.96 (1.90; 4.61) respectively, with little difference when restricted only to APH who ever received ART. In adjusted analyses mortality was similar for male and female APH.

**Conclusions:**

Results highlight probable inequality in mortality according to CIG in SSA even when ART was received. These findings highlight that without attention towards SDG 10 (to reduce inequality within and among countries), progress towards ensuring healthy lives and promoting wellbeing for all at all ages (SDG 3) will be hampered for APH in LIC and LMIC.

## 1 Introduction

Sub‐Saharan Africa (SSA) is a complex region marked by diversity and inequality. Across the continent gross national income per capita varies almost thirty fold from <USD300 to more than USD7,500, national adult literacy rates are as low as 25% in some countries and as high as 96% in others and national under‐5 mortality rates range from <40/1000 to >160/1000 1. Sub‐Saharan Africa is also home to 80% of the 1.8 million adolescents age 10 to 19 years (as defined by the World Health Organisation) living with perinatally or horizontally acquired HIV and 14 of the 15 countries with the highest burden of adolescent HIV 2, 3, 4. Where adolescent‐specific estimates of HIV‐prevalence are available, this ranges in younger adolescents, age 10 to 14 years, from 0.6% in Kenya to almost 3% in Zimbabwe and for older adolescents, age 15 to 19 years from 0.5% in Côte d'Ivoire to 5% in Mozambique 2.

With increasing availability in SSA of early infant diagnosis and antiretroviral therapy (ART), there is now a burgeoning population of adolescents living with perinatally acquired HIV (APH) 5, 6. However, progress in scaling up HIV diagnostic and treatment interventions has not been uniform across the continent. By modelled estimates, coverage of ART in children age 0 to 14 years is only 20% (uncertainty bound (UB) 16%–25%) in West and Central Africa compared to 63% (UB 56%–71%) in East and Southern Africa 3. Furthermore, although AIDS‐related deaths in younger adolescents have started to decline in a number of high‐burden countries, they continue to rise in others 2, 3. Inequality in health and wellbeing for adolescents compared to children and adults living with HIV is evident, with adolescents experiencing greater challenges remaining in care, lower rates of virological suppression and higher rates of mortality 7, 8, 9, 10, 11.

The Collaborative Initiative for Paediatric HIV Education and Research (CIPHER) Cohort Collaboration, previously conducted a global analysis of the epidemiology of APH comparing characteristics and outcomes across multiple regions of the world 12. In this analysis we observed that the hazard for mortality was two to four times higher for APH residing in SSA than for APH in Europe 12. However, as the global community pursues attainment of the United Nations Sustainable Development Goals (SDGs) by 2030, a more precise understanding of how the APH experience differs across SSA and where the inequalities or inequities lie within the region is required. This will aid informing the appropriate global and regional policy response for APH needed to achieve the SDG targets related to ensuring healthy lives and promoting wellbeing for all at all ages (Goal 3), gender equality (Goal 5) and reducing inequality within and between countries (Goal 10) 13.

As such, the primary objective of this CIPHER analysis was to compare the patient and treatment characteristics of APH in SSA by country income groups (CIG), sex and birth cohort. Our secondary objective was to compare the outcomes of mortality, transfer and loss to follow‐up between 10 and 15 years of age across CIG, sex and birth cohort.

## 2 Methods

### 2.1 Study methods

The CIPHER Cohort Collaboration is a global network of observational paediatric HIV cohorts or cohort networks convened by CIPHER of the International AIDS Society, contributed to by 12 cohort networks described elsewhere 12. In this sub‐Saharan Africa‐specific analysis, individual patient‐level data from seven networks was included: Baylor International Pediatric AIDS Initiative at *Texas Children's Hospital* (BIPAI); International Epidemiology Database to Evaluate AIDS (IeDEA) – Central Africa; IeDEA – East Africa; IeDEA – Southern Africa; IeDEA – West Africa; Médecins Sans Frontières Pediatric Cohorts; Identifying Optimal Models for Care in Africa (Optimal Models‐ICAP). The data contributed by the networks were drawn from a range of care settings including routine care cohorts and programmatic services. Twenty two of 56 included cohorts were ART‐only cohorts in which ≥95% of APH received ART; 12/40 cohorts in low‐income countries (LIC), 1/5 cohorts in lower‐middle income countries (LMIC) and 9/11 cohorts in upper‐middle income countries (UMIC).

### 2.2 Analytical methods

This cohort analysis was restricted to APH resident in sub‐Saharan Africa. APH were defined as HIV‐infected children with at least one recorded HIV care visit prior to age 10 years, as a proxy for perinatal HIV infection, and at least one additional HIV care visit after 10 years of age. Children with known non‐vertical routes of HIV infection were excluded.

Our primary analysis described patient characteristics (age, height, weight, CD4 T‐lymphocyte counts and percentages) of APH at key time points including first ever HIV‐associated clinic visit, ART start, age 10 years and last visit (for surviving APH only). These characteristics were compared by CIG, sex and birth cohort. Individual country level characteristics were described for countries with at least 50 APH included (see Supplementary Tables S3 and S4). Observation time was censored at 19 years of age in adolescents with follow‐up beyond this age. World Health Organization (WHO) weight‐for‐age (WAZ) and height‐for‐age Z‐scores (HAZ) were calculated from the measured weights and heights for APH in all regions using the WHO “igrowup_restricted” Stata macro for measurements up to 5 years of age 14 and the “who2007” Stata macro for measurements from age 5–10 years for WAZ and age 5–19 years for HAZ 15. Stunting was defined as HAZ <−2. CIG were assigned according to World Bank country income group classification for the median year of first visit for each country 16. Birth cohorts were classified as born prior to 2000 or born in the year 2000 or later.

Our secondary analysis focused on patient outcomes classified as mortality, transferred out, lost to follow‐up (LTFU) or alive and retained in care. Mortality included all‐cause mortality as reported in the database. Transfer out included documented transfer to a different HIV care site for any reason. LTFU was defined as no observed visit for more than 365 days before the last recorded visit for the cohort. APH classified as LTFU were censored 365 days after their last observed visit. APH considered to be alive and in care at database closure were those not known to have died or transferred and with an observed visit within 365 days prior to the last visit for the cohort. Cumulative incidence functions for the outcomes mortality, transfer out and LTFU at 15 years of age were calculated using competing risks analysis for the whole cohort as well as by CIG, sex and birth period. Transfer out and LTFU were both considered to be competing risks for mortality rather than censoring events. This approach was chosen as the survival distribution of adolescents transferred out or LTFU is likely to be different to those retained in care, with better survival in stable transferred patients and poorer survival in patients LTFU and possibly no longer on ART 17.

Mortality across CIG was further compared by hazard ratios and 95% confidence intervals (CI) using Cox proportional hazard models with delayed entry at age 10 years. Proportionality assumptions were evaluated using the Schoenfeld test. Adjusted hazard ratios were calculated controlling for baseline differences between CIG. Missing CD4, weight and height measurements were imputed for the multivariable models using multiple imputation by chained equations with five iterations of 20 cycles each 18. The imputation model contained all measured variables and used predictive mean matching for anthropometric and CD4 measures. All analyses were conducted using Stata version 13.0 (StataCorp, College Station, Texas, USA) and the “stcompet” package was used to calculate the cumulative incidence functions from the competing risks analysis. Figures were plotted using the ggplot2 package in R version 3.2.2 (R Foundation for Statistical Computing, Vienna, Austria).

Primary data collection by all participating networks was approved by their respective research ethics boards of authority. The pooling of data and analysis at the University of Cape Town (UCT) data centre was approved by the UCT Health Research Ethics Committee [HREC Ref 264/2014].

## 3 Results

This analysis includes 30,296 APH from 25 countries in SSA, 20 countries designated as low income countries (LIC; Table 1). Seventy five percent of APH resided in LIC, compared to 4.6% in LMIC and 19.8% in UMIC. A total of 78,619 years of adolescent follow‐up between age 10 and 19 years were observed.

**Table 1:** Country income group classification of countries represented in sub‐Saharan African CIPHER adolescent cohort

Low income N = 22,925	Lower‐middle income N = 1,386	Upper‐middle income N = 5,985
Benin (N = 44) Burkina Faso (N = 122) Burundi (N = 66) Central African Republic (N = 2) Democratic Republic of Congo (N = 402) Cote d'Ivoire (N = 635) Ethiopia (N = 1,761) Ghana (N = 148) Guinea (N = 75) Kenya (N = 5,913) Malawi (N = 1,753) Mali (N = 208) Mozambique (N = 1,523) Rwanda (N = 1,244) Senegal (N = 88) Tanzania (N = 1,521) Togo (N = 31) Uganda (N = 2,313) Zambia (N = 4,224) Zimbabwe (N = 952)	Cameroon (N = 38) Lesotho (N = 793) Swaziland (N = 555)	Botswana (N = 540) South Africa (N = 5,445)

Countries classified according to World Bank country income group for median year of first visit per country. N represents number of APH included in analysis.

**Figure 1:**
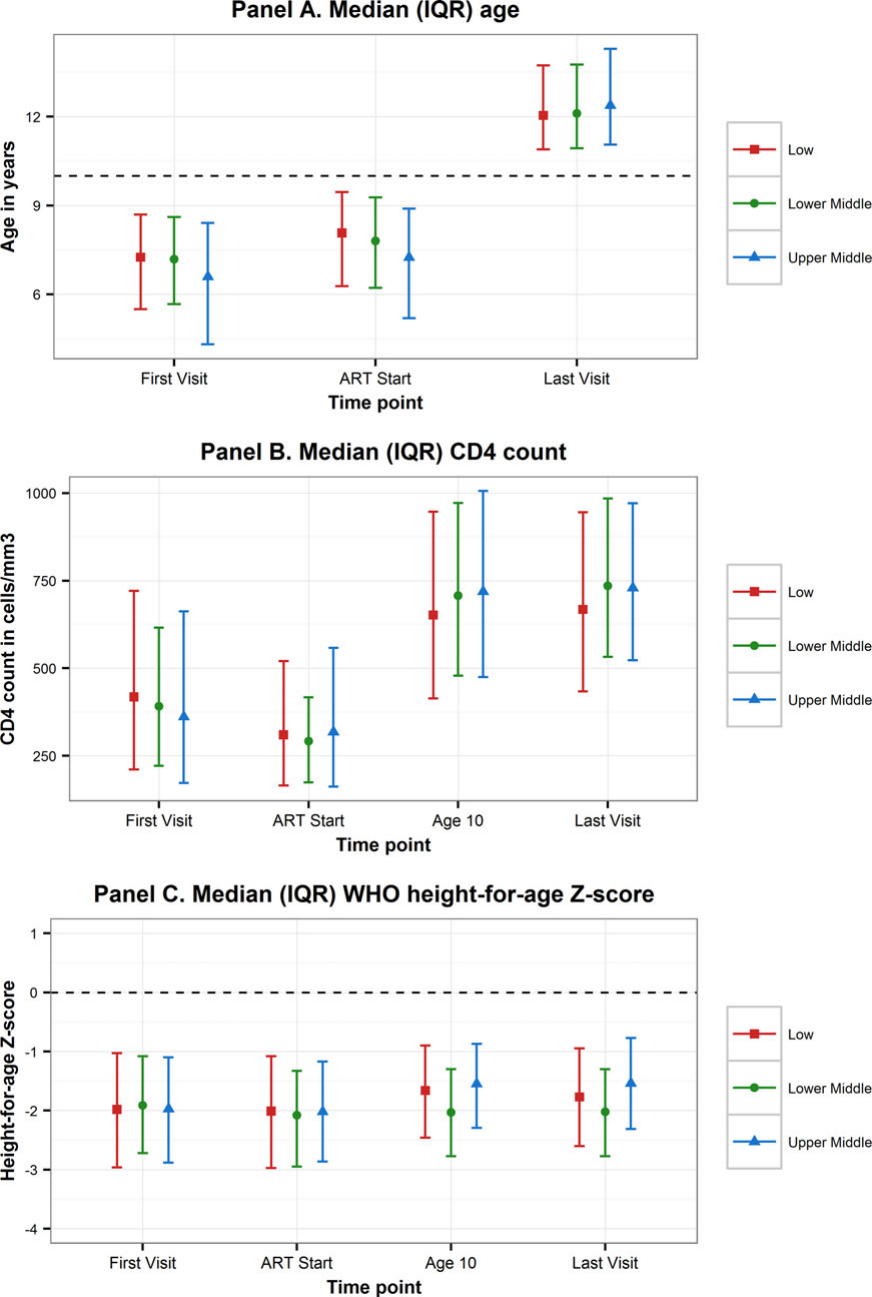
Graphic comparison by country income group of characteristics at first visit, ART start, age 10 years and last visit of adolescents living with perinatally acquired HIV.

For the total cohort, birth year ranged from 1994 to 2005 with the earliest documented HIV‐associated visit occurring in 1996 and follow‐up continuing until at least 2014 in all CIGs. The majority of APH in this cohort were born in the year 2000 or later, 65.4% in LIC, 69.5% in LMIC and 56.9% in UMIC. The median [interquartile range (IQR)] age at first visit was younger in UMIC (6.6 [4.3; 8.4] years) than in LIC (7.3 [5.5; 8.7] years) and LMIC (7.8 [6.2; 8.6] years) as was age at ART start (Table 2, Figure 1). Median [IQR] age at last follow‐up was 12.1 [10.9; 13.8] years with little difference between CIGs. Median [IQR] CD4 count at ART start was 310 [165; 520] cells/mm^3^, with APH in all CIG experiencing a substantial improvement in CD4 count and CD4 percent between ART start and last visit, the largest increase occurring in APH in LMIC (Table 2, Figure 1). Height growth was severely impaired at ART start, with the median HAZ <−2 in all CIGs. APH in LIC and UMIC experienced an improvement in HAZ by last visit, but not APH in LMIC. APH in UMIC experienced the largest improvement in HAZ. In the total cohort, of which 21.5% of APH were in ART‐only cohorts, 88% received ART at some stage. In the 78.5% of APH in cohorts including pre‐ART children 82.8% of APH received ART at some stage. Among APH that did receive ART, 14.3% in LIC only started ART after age 10 years compared to 11.7% in LMIC and 6.6% in UMIC (*p* < 0.0001).

**Table 2:** Adolescent characteristics at first visit, ART start, age 10 years and last visit compared by country income group

	Total	Low income	Lower‐middle income	Upper‐middle income
Total N (row %)	30,296 (100.0)	22,925 (75.7)	1,386 (4.6)	5,985 (19.8)
Male – N (%)	15,007 (49.5)	11,258 (49.1)	697 (50.3)	3,052 (51.0)
Birth Cohort				
Born 2000–2005 – N (%)	19,352 (63.9)	14,982 (65.4)	963 (69.5)	3,407 (56.9)
Year of birth – median (IQR)	2000 (1999; 2002)	2000 (1999; 2002)	2001 (1999; 2002)	2000 (1998; 2002)
Age in years – median (IQR)				
First visit	7.1 (5.3; 8.6)	7.3 (5.5; 8.7)	7.2 (5.7; 8.6)	6.6 (4.3; 8.4)
ART start	7.9 (6.0; 9.3)	8.1 (6.3; 9.5)	7.8 (6.2; 9.3)	7.3 (5.2; 8.9)
Last visit	12.1 (10.9; 13.8)	12.0 (10.9; 13.7)	12.1 (10.9; 13.8)	12.4 (11.1; 14.3)
CD4 count in cells/mm^3^ – median (IQR)				
First visit all ages (N = 15,582)	405 (201; 699)	418 (211; 721)	391 (221; 616)	361 (172; 662)
First visit if age ≥ 5 years (N = 12,591)	370 (180; 646)	388 (191; 678)	370 (208; 592)	296 (134; 521)
ART start all ages (N = 15254)	310 (165; 520)	310 (165; 520)	292 (174; 417)	318 (162; 558)
ART start if age ≥ 5 years (N = 13,635)	301 (158; 500)	309 (163; 526)	285 (168; 380)	281 (139; 474)
Age 10 years (N = 19,829)	671 (430; 964)	652 (414; 947)	707 (479; 973)	719 (475; 1006)
Last visit (N = 24,223)	689 (460; 953)	668 (434; 945)	735 (532; 985)	729 (513; 971)
Mean CD4 count change^a^ (95% CI; N = 15,784)	318 (312; 326)	295 (286; 303)	463 (440; 486)	353 (338; 367)
CD4% – median (IQR)				
First visit (N = 10,201)	15 (9; 23)	16 (10; 25)	14 (9; 21)	14 (8; 20)
ART start (N = 10,386)	13 (8; 18)	13 (8; 19)	12 (7; 17)	13 (8; 18)
Age 10 years (N = 12,089)	27 (20; 34)	27 (19; 34)	28 (21; 35)	28 (20; 34)
Last visit (N = 16,652)	28 (20; 35)	28 (20; 35)	30 (23; 36)	29 (21; 35)
Mean CD4% change^a^ (95% CI; N = 10,483)	14 (13; 14)	13 (12; 13)	18 (17; 19)	14 (14; 15)
WAZ – median (IQR)				
First visit (N = 22,073)	−1.76 (−2.74; −0.90)	−1.81 (−2.80; −0.94)	−1.85 (−2.82; −1.03)	−1.45 (−2.37; −0.64)
ART start (N = 19,658)	−1.75 (−2.70; −0.92)	−1.81 (−2.76; −0.98)	−1.94 (−2.83; −1.10)	−1.46 (−2.36; −0.64)
Age 10 years (N = 24,794)	−1.46 (−2.24; −0.75)	−1.49 (−2.35; −0.80)	−1.54 (−2.27; −0.87)	−1.12 (−1.81; −0.42)
HAZ – median (IQR)				
First visit (N = 16,525)	−1.97 (−2.94; −1.04)	−1.98 (−2.96; −1.03)	−1.91 (−2.72; −1.08)	−1.97 (−2.88; −1.10)
ART start (N = 16,181)	−2.02 (−2.95; −1.11)	−2.01 (−2.97; −1.08)	−2.08 (−2.95; −1.33)	−2.02 (−2.86; −1.17)
Age 10 years (N = 20,584)	−1.66 (−2.45; −0.91)	−1.66 (−2.46; −0.90)	−2.03 (−2.77; −1.30)	−1.55 (−2.29; −0.87)
Last visit (N = 25,333)	−1.75 (−2.57; −0.94)	−1.77 (−2.60; −0.95)	−2.02 (−2.77; −1.30)	−1.54 (−2.31; −0.77)
Mean HAZ change^a^ (95% CI; N = 16,512)	0.20 (0.18; 0.22)	0.16 (0.14; 0.18)	0.04 (−0.02; 0.10)	0.44 (0.40; 0.49)
ART – N (%)				
Ever received	26,727 (88.2)	19,768 (86.3)	1,209 (87.2)	5,750 (96.1)
Started > age 10 years	3,352 (12.9)	2,829 (14.3)	141 (11.7)	382 (6.6)
On ART at age 10 years	19,729 (65.1)	13,919 (60.7)	1,015 (73.2)	4,795 (80.1)
On ART at last visit	23,321 (78.5)	16,744 (74.6)	1,127 (83.4)	5,450 (91.8)

ART, antiretroviral therapy; HAZ, height‐for‐age z‐score; IQR, interquartile range; WAZ, weight‐for‐age *z*‐score.

a Change between antiretroviral therapy start and last visit.

**Figure 2:**
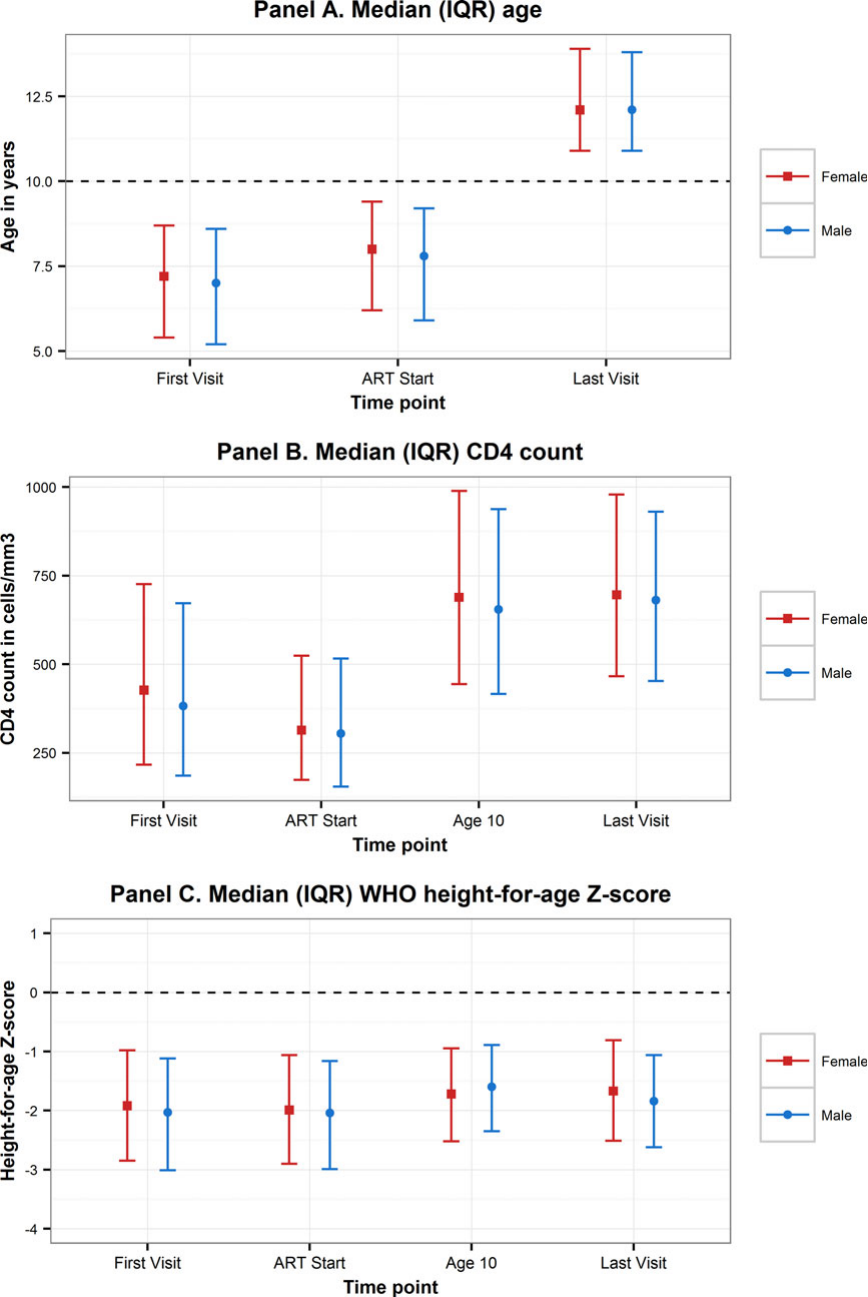
Graphic comparison by sex of characteristics at first visit, ART start, age 10 years and last visit of adolescents living with perinatally acquired HIV.

There were few differences between male and female APH (Table 3, Figure 2). Male APH had a lower CD4 count at first visit and lower absolute CD4 count change than female APH, but equivalent improvement in CD4 percent between ART start and last visit. Male APH experienced less of an improvement than female APH in HAZ between ART start and last visit. A greater proportion of female than male APH started ART after 10 years of age (13.5% vs. 11.6%, *p* < 0.0001).

**Table 3:** Adolescent characteristics at first visit, ART start, age 10 years and last visit compared by sex

	Total	Female	Male
Total N (row %)	30,296 (100.0)	15,289 (50.5)	15,007 (49.5)
Birth cohort – N (%)			
2000–2005	19,352 (63.9)	9,711 (63.5)	9,641 (64.2)
Age in years – median (IQR)			
First visit	7.1 (5.3; 8.6)	7.2 (5.4; 8.7)	7.0 (5.2; 8.6)
ART start	7.9 (6.0; 9.3)	8.0 (6.2; 9.4)	7.8 (5.9; 9.2)
Last visit	12.1 (10.9; 13.8)	12.1 (10.9; 13.9)	12.1 (10.91; 13.8)
CD4 count in cells/mm^3^ – median (IQR)			
First visit all ages (N = 15,582)	405 (201; 699)	427 (217; 726)	382 (186; 672)
First visit if age ≥ 5 years (N = 12,591)	370 (180; 646)	393 (198; 676)	348 (161; 614)
ART start all ages (N = 15,254)	310 (165; 520)	314 (174; 524)	305 (155; 516)
ART start if age ≥ 5 years (N = 13,635)	301 (158; 500)	308 (170; 509)	292 (146; 491)
Age 10 years (N = 19,829)	671 (430; 964)	689 (444; 989)	655 (416; 938)
Last visit (N = 24,223)	689 (460; 953)	696 (466; 979)	681 (453; 931)
Mean CD4 count change^a^ (95% CI; N = 15,784)	318 (312; 326)	329 (319; 339)	308 (299; 318)
CD4% – median (IQR)			
First visit (N = 10,201)	15 (9; 23)	16 (10; 24)	14 (8; 22)
ART start (N = 10,386)	13 (8; 18)	13 (8; 19)	12 (7; 18)
Age 10 years (N = 12,089)	27 (20; 34)	28 (20; 35)	26 (19; 33)
Last visit (N = 16,652)	28 (20; 35)	29 (21; 36)	28 (20; 34)
Mean CD4% change^a^ (95% CI; N = 10,483)	14 (13; 14)	14 (13; 14)	14 (13; 14)
WAZ – median (IQR)			
First visit (N = 22,073)	−1.76 (−2.74; −0.90)	−1.69 (−2.66; −0.85)	−1.82 (−2.84; −0.96)
ART start (N = 19,658)	−1.75 (−2.70; −0.92)	−1.72 (−2.66; −0.90)	−1.78 (−2.75; −0.93)
Age 10 years (N = 24,794)	−1.46 (−2.24; −0.75)	−1.51 (−2.29; −0.78)	−1.44 (−2.17; −0.68)
HAZ – median (IQR)			
First visit (N = 16,525)	−1.97 (−2.94; −1.04)	−1.92 (−2.85; −0.98)	−2.03 (−3.01; −1.12)
ART start (N = 16,181)	−2.02 (−2.95; −1.11)	−1.99 (−2.90; −1.06)	−2.04 (−2.99; −1.16)
Age 10 years (N = 20,584)	−1.66 (−2.45; −0.91)	−1.72 (−2.52; −0.95)	−1.60 (−2.35; −0.89)
Last visit (N = 25,333)	−1.75 (−2.57; −0.94)	−1.67 (−2.51; −0.81)	−1.84 (−2.62; −1.06)
Mean HAZ change^a^ (95% CI; N = 16,512)	0.20 (0.18; 0.22)	0.25 (0.22; 0.28)	0.15 (0.12; 0.18)
ART – N (%)			
Ever received	26,727 (88.2)	13,338 (87.2)	13,389 (89.2)
Started > age 10 years	3,352 (12.9)	1,794 (13.5)	1,558 (11.6)
On ART at age 10 years	19,729 (65.1)	9,690 (63.4)	10,039 (66.9)
On ART at last visit	23,321 (78.5)	11,638 (77.6)	11,683 (79.4)

ART, antiretroviral therapy; HAZ, height‐for‐age *z*‐score; IQR, interquartile range; WAZ, weight‐for‐age *z*‐score.

a Change between antiretroviral therapy start and last visit.

Compared to APH born prior to 2000, age at first visit and ART start was younger for APH in the most recent birth cohort born in 2000 or later (Supplementary Table S1, Supplementary Figure S1). CD4 count at ART start, last visit and CD4 count change between ART start and last visit were all higher in the most recent birth cohort than the birth cohort born prior to 2000. HAZ was severely impaired at first visit and ART start in both birth cohorts, with similar improvements in HAZ by last visit. Similar proportions of APH started ART in both birth cohorts, however a greater proportion started after age 10 years amongst APH born prior to 2000 compared to APH born in 2000 or later (19.8% vs. 8.4%, *p* < 0.0001).

Documented mortality occurred in a total of 576 (1.9%) APH, 3,941 (13.0%) APH were known to have been transferred out for any reason and 2,363 (7.8%) were LTFU. The cumulative incidence [95% CI] in the total cohort for observed mortality between 10 and 15 years of age before any other competing event was 2.92% [2.67; 3.21], ranging from 1.05% [0.75; 1.44] in UMIC to 3.85% [2.67; 5.36] in LMIC and 3.45% [3.12; 3.80] in LIC (Table 4). The cumulative incidence for transfer out between 10 and 15 years of age ranged from 17.54% [16.82; 18.26] in LIC to 27.53% [24.16; 30.99] in LMIC. The cumulative incidence of LTFU was highest in UMIC (14.08% [12.89; 15.33]) and lowest in LMIC (8.27% [6.28; 10.61]). Restricting to only APH that did ever receive ART marginally reduced the cumulative incidence estimates for mortality in all CIG. Stratified by birth cohort, the cumulative incidence for mortality was lower in the most recent birth cohort in all CIG, however LTFU was higher in LIC and UMIC in the most recent birth cohort (Table 4). Stratified by sex, the estimated cumulative incidence for mortality, transfer out and LTFU for the total cohort was similar in males and females. However, in LMIC the cumulative incidence for mortality was higher in males than in females and in UMIC the opposite was observed (Table 4).

**Table 4:** Cumulative incidence of outcomes (mortality, transfer out and lost to follow‐up) between 10 and 15 years of age compared by country income group

	Total	Low income	Lower‐middle income	Upper‐middle income
All adolescents				
N	30,296	22,925	1,386	5,985
Mortality %	2.92 (2.67; 3.21)	3.45 (3.12; 3.80)	3.85 (2.67; 5.36)	1.05 (0.75; 1.44)
Transfer out %	19.34 (18.71; 19.98)	17.54 (16.82; 18.26)	27.53 (24.16; 30.99)	23.72 (22.35; 25.12)
Lost to follow‐up %	13.15 (12.58; 13.73)	13.07 (12.41; 13.75)	8.27 (6.28; 10.61)	14.08 (12.89; 15.33)
Restricted to adolescents ever receiving antiretroviral therapy				
N	26,018	19,114	1,207	5,697
Mortality %	2.86 (2.58; 3.16)	3.43 (3.07; 3.83)	3.91 (2.62; 5.58)	1.02 (0.71; 1.42)
Transfer out %	18.57 (17.90; 19.24)	16.32 (15.56; 17.11)	26.67 (23.07; 30.39)	23.76 (22.36; 25.20)
Lost to follow‐up %	10.39 (9.84; 10.96)	9.98 (9.34; 10.64)	2.94 (1.72; 4.69)	12.62 (11.45; 13.85)
Stratified by birth cohort				
Born < year 2000				
N	10,944	7,943	423	2,578
Mortality %	3.53 (3.17; 3.90)	4.15 (3.71: 4.63)	5.01 (3.20; 7.39)	1.39 (0.97; 1.95)
Transfer out %	16.98 (16.24; 17.74)	15.16 (14.32; 16.02)	22.09 (18.16; 26.27)	21.83 (20.15; 23.56)
Lost to follow‐up %	11.57 (10.93; 12.22)	12.17 (11.41; 12.96)	8.74 (6.22; 11.78)	10.17 (8.93; 11.50)
Born ≥ year 2000				
N	19,352	14,982	963	3,407
Mortality %	2.01 (1.65; 2.44)	2.44 (1.92; 3.06)	4.07 (1.50; 8.69)	0.39 (0.19; 0.74)
Transfer out %	24.53 (22.58; 26.51)	22.41 (20.18; 24.73)	89.43 (84.05; 93.08)	25.34 (22.72; 28.02)
Lost to follow‐up %	23.03 (19.70; 26.53)	21.31 (17.13; 25.81)	6.50 (4.10; 9.64)	25.11 (21.30; 29.08)
Stratified by sex				
Female				
N	15,289	11,667	689	2,933
Mortality %	2.90 (2.54; 3.30)	3.35 (2.90; 3.84)	2.07 (1.08; 3.61)	1.34 (0.87; 2.00)
Transfer out %	18.68 (17.82; 19.55)	16.94 (15.97; 17.94)	24.70 (20.49; 29.13)	23.51 (21.57; 25.49)
Lost to follow‐up %	13.32 (12.52; 14.15)	13.28 (12.35; 14.24)	8.45 (5.72; 11.90)	14.06 (12.37; 15.85)
Male				
N	15,007	11,258	697	3,052
Mortality %	2.93 (2.57; 3.32)	3.51 (3.04; 4.03)	4.86 (3.04; 7.30)	0.60 (0.34; 0.99)
Transfer out %	20.04 (19.11; 20.98)	18.14 (17.08; 19.23)	29.63 (24.61; 34.81)	23.83 (21.90; 25.82)
Lost to follow‐up %	12.97 (12.18; 13.79)	12.80 (11.8; 13.76)	7.47 (4.99; 10.60)	14.01 (12.36; 15.76)

The hazard of observed mortality was substantially higher in APH in LIC (adjusted hazard ratio (aHR) 3.05 [95% CI 2.27; 4.09]) and LMIC (aHR 3.57 [2.30; 5.54]) compared to UMIC (Table 5, Supplementary Table S2, model 1). Adjusting for differences in baseline characteristics across CIG including sex, birth cohort, ever on ART and first visit‐age, ‐CD4 count, ‐WAZ and ‐HAZ, marginally increased the aHR for mortality in APH in LIC and LMIC relative to UMIC in a model including complete cases only (Table 5 and Supplementary Table S2, model 2) and somewhat reduced the aHR in a model including all cases with imputed missing values (Table 5 and Supplementary Table S2, model 3). In a model restricted only to APH that did ever receive ART, observed mortality remained substantially elevated for APH in LIC (aHR 2.67 [1.94; 3.67]) and LMIC (aHR 3.07 [1.91; 4.95]), relative to APH in UMIC (Table 5 and Supplementary Table S2, model 4). After controlling for baseline differences there was no significant effect of sex on mortality nor was there a significant interaction between sex and CIG or sex and age at first visit or ART start.

**Table 5:** Mortality hazard ratios (95% confidence intervals) by country income group with reference to upper‐middle income countries

Low income	Lower‐middle income	Upper‐middle income
1. Unadjusted HR (N = 30,296)		
3.05 (2.27; 4.09)	3.57 (2.30; 5.54)	Reference
2. Adjusted^a^ HR – complete cases only (N = 13,985)		
3.75 (2.02; 6.95)	3.74 (1.80; 7.78)	Reference
3. Adjusted^a^ HR – multiple imputation for missing CD4, WAZ, HAZ (N = 30,296)		
2.50 (1.85; 3.37)	2.96 (1.90; 4.61)	Reference
4. Adjusted HR^b^ – multiple imputation for missing CD4, WAZ, HAZ & restricted to those ever on ART (N = 26,018)		
2.67 (1.94; 3.67)	3.07 (1.91; 4.95)	Reference

ART, antiretroviral therapy; HAZ, height‐for‐age Z‐score; HR, hazard ratio; WAZ, weight‐for‐age *z*‐score.

a Adjusted for – on ART ever; sex; age; birth cohort; first visit ‐CD4 count, ‐WAZ, ‐HAZ.

b Adjusted for – sex; age; birth cohort; first visit ‐CD4 count, ‐WAZ, ‐HAZ.

Individual country level description is presented for key characteristics and mortality hazard ratios in Supplementary Tables S3 and S4.

## 4 Discussion

This large cohort describes the characteristics and outcomes of more than 30,000 APH in SSA with almost 80,000 years of combined adolescent follow‐up, three quarters of whom were residing in countries classified as low income. Although this is largely a young adolescent cohort with almost two‐thirds born in 2000 or later, the majority of these APH started ART well into childhood at a median age of almost 8 years and were stunted by the time ART was initiated. Despite this, overall APH experienced improvements following ART start in CD4 measures as well as height. These improvements did differ by CIG though. APH in LMIC experienced the largest improvement in CD4 count and percent, but no improvement in height, whereas APH in UMIC experienced a substantially larger improvement in height growth following ART start than APH in LIC and LMIC. Furthermore, APH in LIC and LMIC experienced at least a three times greater hazard of observed mortality compared to APH in UMIC with this inequality persisting after controlling for baseline differences and when comparing only APH that did ever receive ART.

Considering that the median year of birth for this cohort was the year 2000, at least 5 years before extensive scale‐up of paediatric ART services commenced in SSA 19, and that the median age at first visit was 7 years, this cohort of APH likely represents the best‐case scenario for the current generation of APH in SSA. A much larger cohort of children in SSA would have died prior to being diagnosed, linked to HIV care or reaching 10 years of age 20. In this context, amongst APH that did receive ART, even though age and CD4 measures at ART start did not differ markedly between CIG, improvement in height growth was substantially better and mortality substantially lower in APH from UMIC than LIC and LMIC. Impaired length growth during childhood, specifically stunting, is associated with numerous detrimental consequences that can impact on social and economic functioning during adolescence and adulthood including poorer educational grade attainment, lower adult income and reduced likelihood of gaining formal adult employment 21, 22, 23. In light of this, neurocognitive outcomes and other morbidities, not measured in this cohort, could also be expected to be worse in APH in lower income settings 9. Thus, despite receiving HIV care and treatment, inequality remains in survival, health and wellbeing of APH in LIC and LMIC compared to UMIC. These inequalities in growth and survival indicate how progress towards SDG 3, specifically reducing HIV‐associated mortality during adolescence, is intertwined with SDG 10 to reduce inequality within and between countries.

In male APH a smaller improvement in height growth was observed after starting ART than in female APH. This is consistent with previous studies in HIV‐infected as well as HIV‐uninfected children that have observed greater impairments in length growth in male than female children 24, 25. In this cohort of adolescents with median follow‐up to 12 years of age, the timing of the pubertal growth spurt may account for some of this difference. Puberty is known to be delayed in HIV‐infected children and females experience accelerated pubertal growth earlier than males with the potential for males to recover this growth deficit later in adolescence 26, 27. Although in the descriptive and unadjusted analysis there appeared to be differences in mortality between male and female APH, these did not remain in the adjusted analyses. In this cohort male and female APH had a similar hazard of mortality with no evidence for gender inequality in mortality up to age 15 years.

Until recently, national and global HIV monitoring systems have largely ignored the adolescent age group with the only age‐disaggregation of indicators being above (adult) and below (children) 15 years of age. UNAIDS now reports adolescent specific HIV indicators where possible and in 2015 the World Health Organization issued Consolidated Strategic Information Guidelines that strongly recommend age disaggregation in 5 year age bands 3, 28, 29. With expanding electronic monitoring systems in SSA such age disaggregation is becoming feasible 30, however challenges remain for countries even with electronic monitoring systems to provide appropriately age‐disaggregated data 31. Particularly in SSA with high rates of maternal and child mortality, national health information systems have not been oriented towards monitoring the general adolescent population, who with lower all‐cause mortality rates than other age groups, receive little attention from healthcare systems 32, 33. Efforts towards SDG 17.18, to increase significantly the availability of high‐quality, timely and reliable data disaggregated by income, sex and age among other parameters, have the potential to greatly improve monitoring of health outcomes for all adolescents and specifically adolescents living with HIV.

We recognize that classifying countries according to income groups at a single point in time represents a unidimensional, static assessment of their capacity and that achievements in accelerating health and development are influenced by governmental, legal, societal and numerous other structures not represented by the CIG classification 11. Furthermore, the CIG of the country may not correspond with individual household level income, the effect of which we are not able to address with this dataset and in this analysis. However, comparing outcomes by CIG is a first step to more broadly understanding how outcomes for APH in SSA may be influenced by factors beyond the healthcare system and provision of ART. This analysis is not able to interrogate what the socio‐economic and structural drivers of mortality in APH are, however it does highlight the need for studies that can inform socio‐economic and structural interventions to improve health and survival of APH across SSA. Moreover, with indications that international funding for HIV is likely to plateau, and evidence that most low income HIV high‐burden countries are unlikely to have the domestic capacity to finance the needs of their HIV‐epidemic, it is appropriate to consider the real possibility that inequalities in outcomes may widen further for APH living in LIC and LMIC compared to UMIC 34.

Our analysis was restricted to adolescents most likely to have perinatally acquired HIV who survived into adolescence and does not apply to the larger population acquiring HIV during adolescence in whom important gender inequalities and additional vulnerabilities exist 35. Due to generally poor recording of mode of transmission, we utilized a pragmatic definition of perinatally acquired HIV according to entry into care before age 10 years, that excluded the important group of APH that are only identified and diagnosed after age 10 years 6. Although our analysis includes representation from 14 of the 15 countries with the highest adolescent HIV burden, it does not include Nigeria, the country with the second largest population of adolescents living with HIV and the only country in which mortality in younger adolescents is still estimated to be increasing 3. This analysis likely overestimates the proportion of APH ever receiving ART, particularly in UMIC where more than 80% of cohorts were ART‐only cohorts compared to <30% of cohorts in LIC and LMIC. With the high rates of LTFU in this cohort, mortality is likely underestimated with a proportion of LTFU due to unascertained mortality. This limits comparison of mortality estimates across CIG. Methods in adult HIV cohort research have been advanced through tracing studies of patients LTFU or linkage to mortality registries to be able to informatively adjust estimates of mortality based on the proportion lost to follow‐up and the size of the ART programme 36, 37. Such methods are yet to be developed for children and adolescents living with HIV, highlighting further inequality in research investment for children and adolescents compared to adults.

## 5 Conclusions

Irrespective of CIG, this cohort of APH entered care and started ART well into childhood, with consequent marked growth impairment likely to impact on social and economic capacity as this generation of adolescents enter adulthood. Even when receiving ART, inferior growth improvement and higher mortality was observed in APH from LIC and LMIC compared to UMIC signalling the role of factors beyond the ART programme in determining the health and wellbeing of APH. Without broader national capacity development in LIC and LMIC in SSA, and measurable progress towards reducing inequality within and among countries (SDG 10), outcomes for APH in LIC and LMIC in SSA will continue to lag behind those of their peers in UMIC. Without concerted efforts in relation to SDG 17.18 to monitor APH within national health information systems, the needs of this diverse and complex population will continue to go unnoticed.

## Competing interests

MVs work at CIPHER is funded through Unrestricted Educational grants received from ViiV Healthcare and Janssen to the International AIDS Society. CS has received personal payment for preparation of educational materials for Gilead Sciences and ViiV Healthcare. JW's institution has received academic grants from the INSERM‐ANRS, for cohorts of JW's responsibility involved in the study. SW receives a fee from Baylor International Pediatric AIDS Initiative for consultancy services related to research. All remaining authors declare no conflicts of interest.

## Authors' contributions

Project Team: Amy L. Slogrove, University of Cape Town, South Africa (project co‐chair, data curation, formal analysis, methodology, visualization, writing – original draft preparation); Marcel Yotebieng, College of Public Health, Ohio State University, USA (project co‐chair, conceptualization, writing – review & editing); Michael Schomaker, University of Cape Town, South Africa (conceptualization, data curation, formal analysis, methodology, resources, software, supervision, validation, visualization, writing – review & editing); Mary‐Ann Davies, University of Cape Town, South Africa (conceptualization, data curation, project administration, supervision, writing – review & editing); Ali Judd, MRC Clinical Trials Unit at University College London, London, UK (conceptualization, project administration, supervision, writing – review & editing); Valériane Leroy, Inserm, U1027, Université Toulouse 3, France (conceptualization, supervision, writing – review & editing); Paige Williams, Harvard T. H. Chan School of Public Health, USA (conceptualization, supervision, writing – review & editing); Suna Balkan, Médecins Sans Frontières Medical Department, France (conceptualization, supervision, writing – review & editing); Jihane Ben‐Farhat, Epicentre, Médecins Sans Frontières, France (conceptualization, supervision, writing – review & editing); Nancy Calles, Baylor International Pediatric AIDS Initiative *at Texas Children's Hospital*‐USA, USA (conceptualization, writing – review & editing); Kulkanya Chokephaibulkit, Siriraj Hospital, Mahidol University, Bangkok, Thailand (investigation, writing – review & editing); Charlotte Duff, MRC Clinical Trials Unit at University College London, London, UK (conceptualization, data curation, writing – review & editing); Tonah François Eboua, CHU Yopougon, Côte d'Ivoire (conceptualization, writing – review & editing); Adeodata Kekitiinwa, Baylor International Pediatric AIDS Initiative *at Texas Children's Hospital* ‐Uganda, Uganda (conceptualization, writing – review & editing); Nicky Maxwell, University of Cape Town, South Africa (conceptualization, data curation, writing – review & editing); Jorge Pinto, School of Medicine, Federal University of Minas Gerais, Brazil (conceptualization, writing – review & editing); George Seage III, Harvard T. H. Chan School of Public Health, USA (conceptualization, project administration, supervision, writing – review & editing); Chloe Teasdale, ICAP‐Columbia University, Mailman School of Public Health, USA (conceptualization, data curation, writing – review & editing); Sebastian Wanless, Baylor International Pediatric AIDS Initiative *at Texas Children's Hospital*‐USA, USA (conceptualization, data curation, writing – review & editing); Josiane Warszawski, French Institute of Health and Medical Research, France (conceptualization, writing – review & editing); Kara Wools‐Kaloustian, Indiana University School of Medicine, USA (conceptualization, supervision, writing – review & editing);

Project Oversight Group: CIPHER Cohort Collaboration Data Centre at Centre for Infectious Disease Epidemiology and Research, University of Cape Town, South Africa ‐ Mary‐Ann Davies, (conceptualization, data curation, project administration, supervision, writing – review & editing); Nicky Maxwell (conceptualization, data curation, writing – review & editing); Michael Schomaker (conceptualization, formal analysis, supervision, writing – review & editing); Venessa Timmerman, (data curation, writing – review & editing); CIPHER Post‐doctoral grantee – Amy L. Slogrove, Centre for Infectious Disease Epidemiology and Research University of Cape Town, South Africa (data curation, formal analysis, writing – original draft preparation); EPPICC – Jeannie Collins, MRC Clinical Trials Unit at University College London, London, UK (supervision, writing – review & editing); Charlotte Duff, MRC Clinical Trials Unit at University College London, London, UK (data curation, writing – review & editing); Ruth Goodall, MRC Clinical Trials Unit at University College London, London, UK (supervision, writing – review & editing); Ali Judd, MRC Clinical Trials Unit at University College London, London, UK (conceptualization, project administration, supervision, writing – review & editing); Colette Smith, Institute of Global Health, University College London, London, UK (supervision, writing – review & editing); IeDEA East Africa ‐ Kara Wools‐Kaloustian, Indiana University School of Medicine, USA (supervision, writing – review & editing); IeDEA West Africa – Valériane Leroy, Inserm, U1027, Université Toulouse 3, France (conceptualization, supervision, writing – review & editing); PHACS/IMPAACT ‐ Kunjal Patel, Harvard T. H. Chan School of Public Health, USA (supervision, writing – review & editing); George Seage III, Harvard School of Public Health, USA (conceptualization, project administration, supervision, writing – review & editing); Paige Williams, Harvard T. H. Chan School of Public Health, USA (conceptualization, supervision, writing – review & editing).

CIPHER Cohort Collaboration Steering Committee: BIPAI ‐ Mary Paul, Baylor International Pediatric AIDS Initiative *at Texas Children's Hospital*, USA (supervision, writing – review & editing); EPPICC ‐ Diana Gibb, MRC Clinical Trials Unit at University College London, London, UK (supervision, writing – review & editing); Ali Judd, MRC Clinical Trials Unit at University College London, London, UK (conceptualization, project administration, supervision, writing – review & editing); IeDEA Southern Africa ‐ Mary‐Ann Davies, University of Cape Town, South Africa (conceptualization, data curation, project administration, supervision, writing – review & editing); IeDEA‐East Africa ‐ Rachel Vreeman, Indiana University (supervision, writing – review & editing); Médecins Sans Frontières ‐ Suna Balkan, MSF Medical Department, France (conceptualization, supervision, writing – review & editing); Jihane Ben‐Farhat, Epicentre, MSF, France (conceptualization, supervision, writing – review & editing); Optimal Models (ICAP) ‐ Elaine Abrams, ICAP‐Columbia University, Mailman School of Public Health, USA (supervision, writing – review & editing); PHACS/IMPAACT ‐ Rohan Hazra, US National Institutes of Health, NICHD, USA (supervision, writing – review & editing); George Seage III, Harvard T. H. Chan School of Public Health, USA (conceptualization, project administration, supervision, writing – review & editing); Russell Van Dyke, Tulane University, USA (supervision, writing – review & editing).

CIPHER Executive Committee: Linda‐Gail Bekker, Desmond Tutu HIV Centre, University of Cape Town, South Africa (supervision, writing – review & editing); Lynne Mofenson, Elizabeth Glaser Pediatric AIDS Foundation, USA (supervision, writing – review & editing); Marissa Vicari, International AIDS Society, Switzerland (funding acquisition, project administration, supervision, writing – review & editing); Shaffiq Essajee, World Health Organization, Switzerland (supervision, writing – review & editing); Martina Penazzato, World Health Organization, Switzerland (supervision, writing – review & editing).

**Representatives of contributing networks**

Baylor International Pediatric AIDS Initiative *at Texas Children's* Hospital: Botswana, Gabriel Anabwani (investigation, writing – review & editing); Lesotho, Edith Q. Mohapi (investigation, writing – review & editing); Malawi, Peter N. Kazembe (investigation, writing – review & editing); Swaziland, Makhosazana Hlatshwayo (investigation, writing – review & editing); Tanzania, Mwita Lumumba (investigation, writing – review & editing); Uganda, Adeodata Kekitiinwa‐Rukyalekere (investigation, writing – review & editing; Data Manager ‐ Sebastien Wanless (conceptualization, data curation, writing – review & editing).

IeDEA Central Africa: Marcel Yotebieng, College of Public Health, Ohio State University, Columbus, USA (conceptualization, investigation, writing – review & editing); Andrew Edmonds, The Gillings School of Public Health, University of North Carolina at Chapel Hill, USA (investigation, writing – review & editing); Patricia Lelo, Pediatric Hospital Kalembe Lembe, Lingwala, Kinshasa, Demogratic Republic of Congo (investigation, writing – review & editing).

IeDEA East Africa: Samuel Ayaya, Academic Model Providing Access to Healthcare (AMPATH), Eldoret, Kenya (investigation, writing – review & editing); Patricia Ongwen, Family AIDS Care and Education Services, Kenya Medical Resarch Institute, Kisumu, Kenya (investigation, writing – review & editing); Rachel Vreeman, Indiana University School of Medicine, Department of Pediatrics, IU Center for Global Health, Indianapolis, Indiana (supervision, writing – review & editing); Kara Wools‐Kaloustian, Indiana University School of Medicine, Department of Medicine, Division of Infectious Diseases, Indianapolis, Indiana (supervision, writing – review & editing).

IeDEA Southern Africa: Carolyn Bolton‐Moore, Centre for Infectious Disease Research in Zambia, Lusaka, Zambia (investigation, writing – review & editing); Frank Tanser, Africa Centre for Population Health, School of Nursing and Public Health and Centre for the AIDS Programme of Research in South Africa (CAPRISA), University of KwaZulu‐Natal, South Africa (investigation, writing – review & editing); Gill Sorour, Empilweni Service and Research Unit, Rahima Moosa Mother and Child Hospital and University of Witwatersrand, Johannesburg, South Africa (investigation, writing – review & editing); Catrina Mugglin, Institute for Social and Preventive Medicine, University of Bern, Switzerland.

IeDEA West Africa: Tanoh Francois Eboua, Yopougon University Hospital, University Félix Houphouët‐Boigny, Abidjan, Ivory Coast (investigation, writing – review & editing); Lorna Renner, Korle Bu Teaching Hospital, Accra, Ghana (investigation, writing – review & editing); Mariam Sylla, CHU Gabriel Touré, Bamako, Mali (investigation, writing – review & editing).

Médecins Sans Frontières: Suna Balkan, France (conceptualization, supervision, writing – review & editing); Jihane Ben‐Farhat, France (conceptualization, supervision, writing – review & editing)

Optimal Models/ICAP: Elaine Abrams, ICAP‐Columbia University, Mailman School of Public Health, USA; Chloe Teasdale, ICAP‐Columbia University, Mailman School of Public Health, USA

## Supporting information


**Table S1** Adolescent characteristics at first visit, ART start, age 10 years and last visit compared by birth cohort.
**Table S2** Complete multivariable models: mortality hazard ratios and 95% confidence intervals.
**Table S3** Individual country descriptive characteristics.
**Table S4** Individual country mortality hazard ratios.

**Figure S1** Graphic comparison by birth cohort of characteristics at first visit, ART start, age 10 years and last visit of adolescents living with perinatally acquired HIV.
