# Supplementary material for: Inequality in outcomes for adolescents living with perinatally acquired HIV in sub‐Saharan Africa: a Collaborative Initiative for Paediatric HIV Education and Research (CIPHER) Cohort Collaboration analysis
Source: J Int AIDS Soc. 2018 Feb 27;21(Suppl Suppl 1):e25044. doi: 10.1002/jia2.25044 (PMC5978669; doi:10.1002/jia2.25044)
Supplement: Supplementary file 1 — Table S1 Adolescent characteristics at first visit, ART start, age 10 years and last visit compared by birth cohort. Table S2 Complete multivariable models: mortality hazard ratios and 95% confidence intervals. Table S3 Individual country descriptive characteristics. Table S4 Individual country mortality hazard ratios. [file JIA2-21-e25044-s001.pdf]

Supplementary Figure 1: Graphic comparison by birth cohort of characteristics at first visit, ART start, age 10 years and last visit of adolescents living with perinatally-acquired HIV

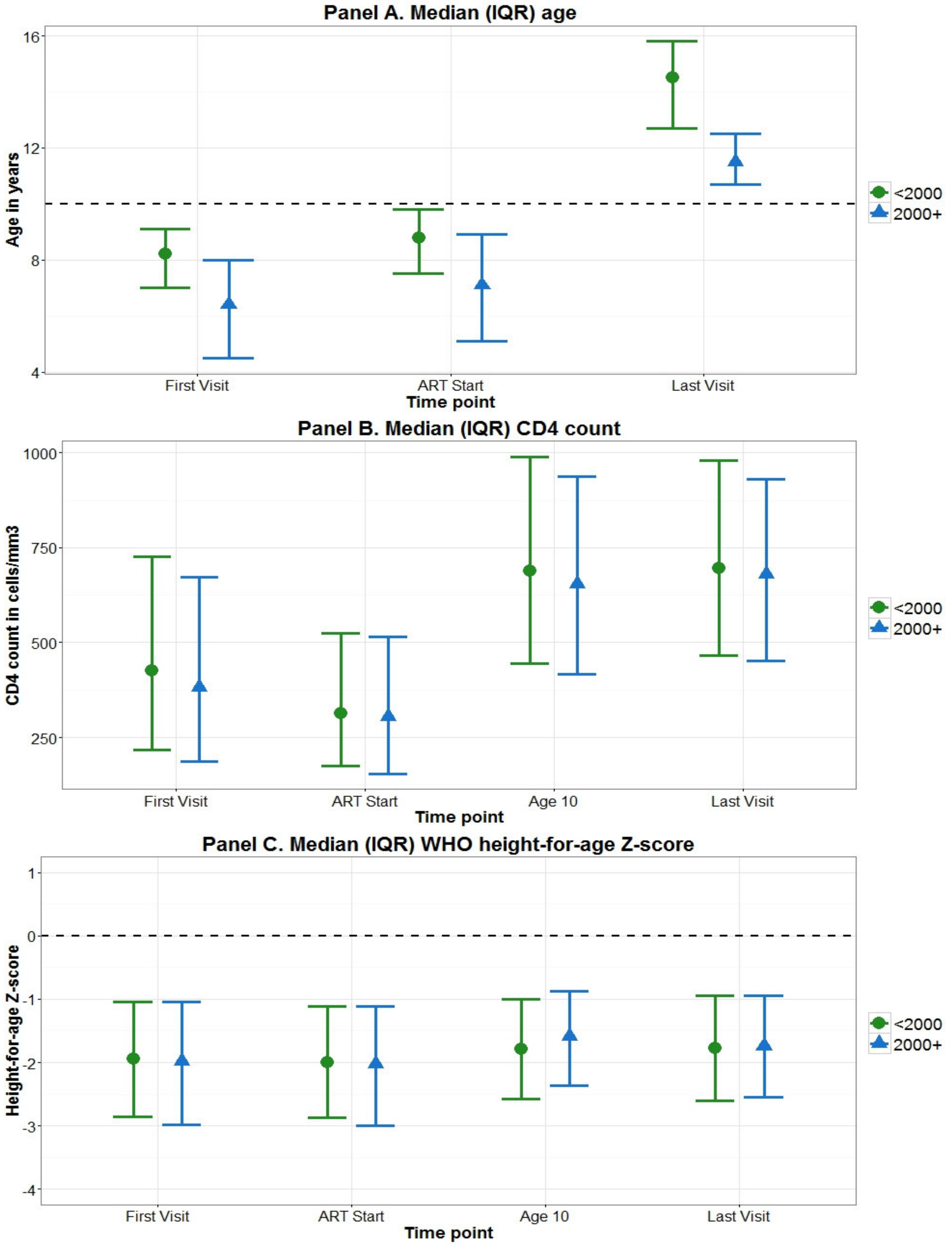
Panel A. Median (IQR) age
Age in years
16
12
8
4
First Visit
ART Start
Last Visit
Time point
<2000
2000+
Panel B. Median (IQR) CD4 count
CD4 count in cells/mm3
1000
750
500
250
First Visit
ART Start
Age 10
Last Visit
Time point
<2000
2000+
Panel C. Median (IQR) WHO height-for-age Z-score
Height-for-age Z-score
1
0
-1
-2
-3
-4
First Visit
ART Start
Age 10
Last Visit
Time point
<2000
2000+

Supplementary Table 1: Adolescent characteristics at first visit, ART start, age 10 years and last visit compared by birth cohort

	Total	Born < year 2000	Born ≥ year 2000
**Total N** (row %)	30,296 (100.0)	10,944 (36.1)	19,352 (63.9)
**Male** – N (%)	15,007 (49.5)	5,366 (49.0)	9,641 (49.8)
**Age in years** – median (IQR)			
First visit	7.1 (5.3; 8.6)	8.2 (7.0; 9.1)	6.4 (4.5; 8.0)
ART start	7.9 (6.0; 9.3)	8.8 (7.5; 9.8)	7.1 (5.1; 8.9)
Last visit	12.1 (10.9; 13.8)	14.5 (12.7; 15.8)	11.5 (10.7; 12.5)
**CD4 count in cells/mm^3** – median (IQR)			
First visit all ages [N=15,582]	405 (201; 699)	330 (147; 570)	457 (240; 765)
First visit if age ≥ 5 years [N=12,591]	370 (180; 646)	325 (143; 564)	401 (214; 696)
ART start all ages [N=15,254]	310 (165; 520)	264 (130; 425)	338 (191; 583)
ART start if age ≥ 5 years [N=13,635]	301 (158; 500)	268 (131; 435)	325 (182; 554)
Age 10 years [N=19,829]	671 (430; 964)	585 (360; 860)	724 (479; 1019)
Last visit [N=24,223]	689 (460; 953)	615 (404; 858)	734 (495; 1006)
Mean CD4 count change* (95% CI) [N=15,784]	318 (312; 326)	311 (301; 321)	324 (315; 333)
**CD4 %** - median (IQR)			
First visit [N=10,201]	15 (9; 23)	14 (8; 21)	16 (10; 24)
ART start [N=10,386]	13 (8; 18)	12 (6; 17)	14 (9; 19)
Age 10 years [N=12,089]	27 (20; 34)	24 (17; 31)	30 (22; 36)
Last visit [N=16,652]	28 (20; 35)	27(19; 33)	29 (21; 36)
Mean CD4 % change* (95% CI) [N=10,483]	13.6 (13.4; 13.8)	13.1 (12.7; 13.4)	13.9 (13.6; 14.2)
**WAZ** – median (IQR)			
First visit	-1.76 (-2.74; -0.90)	-1.86 (-2.82; -1.01)	-1.70 (-2.70; -0.85)

	Total	Born < year 2000	Born ≥ year 2000
[N=22,073]			
ART start	-1.75 (-2.70; -0.92)	-1.87 (-2.82; -1.03)	-1.68 (-2.63; -0.86)
[N=19,658]			
Age 10 years	-1.46 (-2.24; -0.75)	-1.50 (-2.37; -0.78)	-1.45 (-2.14; -0.72)
[N=24,794]			
**HAZ** – median (IQR)			
First visit	-1.97 (-2.94; -1.04)	-1.95 (-2.86; -1.04)	-1.98 (-2.99; -1.04)
[N=16,525]			
ART start	-2.02 (-2.95; -1.11)	-2.00 (-2.87; -1.12)	-2.02 (-3.00 ; -1.11)
[N=16,181]			
Age 10 years	-1.66 (-2.45; -0.91)	-1.79 (-2.57; -1.00)	-1.59 (-2.37; -0.87)
[N=20,584]			
Last visit	-1.75 (-2.57; -0.94)	-1.77 (-2.60; -0.94)	-1.74 (-2.55; -0.94)
[N=25,333]			
Mean HAZ change* (95% CI) [N=16,512]	0.20 (0.18; 0.22)	0.14 (0.11; 0.17)	0.24 (0.21; 0.27)
**ART** – N (%)			
Ever received	26,727 (88.2)	9,823 (89.8)	16,904 (87.4)
Started > age 10 years	3,352 (12.9)	1,927 (19.6)	1,425 (8.4)
On ART at age 10 years	19,729 (65.1)	6,649 (60.8)	13,080 (67.6)
On ART at last visit	23,321 (78.5)	8,508 (80.6)	14,813 (77.3)

ART – antiretroviral therapy; HAZ – height-for-age z-score; IQR – interquartile range; WAZ – weight-for-age z-score:

* Change between antiretroviral therapy start and last visit

Supplementary Table 2: Complete multivariable models: mortality hazard ratios and 95% confidence intervals

	**Model 1: Unadjusted HR**	**Model 2*: Adjusted HR** (complete cases)	**Model 3*: Adjusted HR** (with MI)	**Model 4*: Adjusted HR** restricted to those ever on ART (with MI)
Country Income Group				
Low	3.05 (2.72; 4.09)	3.75 (2.02; 6.95)	2.50 (1.85; 3.37)	2.67 (1.94; 3.67)
Lower-Middle	3.57 (2.30; 5.54)	3.74 (1.80; 7.78)	2.96 (1.90; 4.61)	3.07 (1.91; 4.95)

Upper-Middle	Reference	Reference	Reference	Reference
Sex				
Male	1.06 (0.90; 1.25)	0.89 (0.68; 1.17)	1.03 (0.88; 1.22)	1.10 (0.92; 1.32)
Female	Reference	Reference	Reference	Reference
Age at first visit – per year	1.24 (1.18; 1.30)	1.09 (0.99; 1.20)	1.10 (1.04; 1.16)	1.11 (1.04; 1.18)
On ART				
Ever	0.58 (0.46; 0.72)	0.38 (0.26; 0.57)	0.47 (0.36; 0.60)	NA
Never	Reference	Reference	Reference	NA
Birth Cohort				
< 2000	Reference	Reference	Reference	Reference
≥ year 2000	0.60 (0.50; 0.72)	0.70 (0.50; 0.98)	0.70 (058; 0.86)	0.82 (0.66; 1.02)
CD4 count at first visit – per 50 cells/µl	0.94 (0.92; 0.96)	0.94 (0.92; 0.97)	0.95 (0.94; 0.97)	0.96 (0.93; 0.98)
WAZ at first visit – per 1 Z-score	0.80 (0.75; 0.86)	0.87 (0.76; 0.99)	0.84 (0.77; 0.90)	0.83 (0.76; 0.92)
HAZ at first visit – per 1 Z-score	0.94 (0.88; 1.01)	0.99 (0.89; 1.10)	1.02 (0.94; 1.12)	1.05 (0.94; 1.17)

ART – antiretroviral therapy; HAZ – height-for-age Z-score; HR – hazard ratio; MI – multiple imputation; WAZ – weight-for-age Z-score

* Adjusted for other covariates in the table

Supplementary Table 3: Individual country descriptive characteristics

Country	N	GNI[#]	Health Spending[#]	ART Start			Last Visit			Ever on ART
				Age	CD4 count	HAZ	Age	CD4 count	HAZ	
**Upper Middle Income**										
South Africa	5445	5940	837	7.4 (5.4; 9.0)	310 (154; 536)	-2.03 (-2.89; -1.19)	12.2 (11.0; 14.1)	718 (503; 963)	-1.60 (-2.37; -0.80)	5176 (95.1)
Botswana	540	5620	580	5.3 (2.4; 7.7)	509 (274; 838)	-1.90 (-2.74; -1.04)	14.0 (12.2; 16.4)	813 (582; 1023)	-1.29 (-2.01; -0.63)	521 (96.5)
**Lower Middle Income**										
Swaziland	555	3190	388	7.8 (6.2; 9.3)	303 (187; 442)	-1.76 (-2.69; -0.87)	12.2 (10.9; 13.8)	771 (539; 1009)	-1.63 (-2.34; -0.91)	474 (85.4)
Lesotho	793	1310	155	7.8 (6.3; 9.2)	291 (172; 405)	-2.26 (-3.06; -1.49)	12.1 (10.9; 13.7)	730 (535; 967)	-2.34 (-3.07; -1.60)	702 (88.5)
**Low Income**										
Cote d'Ivoire	635	970	161	7.7 (5.8; 9.1)	411 (202; 649)	-1.57 (-2.40; -0.82)	12.1 (10.9; 13.9)	609 (329; 878)	-1.35 (-2.17; -0.44)	562 (88.5)
Uganda	2313	370	155	7.7 (5.3; 9.5)	276 (137; 457)	-1.96 (-2.95; -0.96)	12.8 (11.2; 14.9)	706 (485; 985)	-1.66 (-2.45; -0.89)	1789 (77.4)
Ghana	148	800	137	7.7 (6.1; 9.0)	301 (67; 535)	-1.82 (-2.94; -0.94)	11.8 (10.9; 12.9)	863 (610; 1158)	-1.41 (-2.14; -0.55)	136 (91.9)
Zambia	4224	880	113	8.0 (6.2; 9.3)	not available	-2.16 (-3.07; -1.29)	12.4 (11.0; 14.2)	not available	-1.79 (-2.65; -0.99)	3899 (92.3)
Mali	208	540	96	8.0 (5.4; 9.5)	267 (116; 415)	-1.65 (-2.67; -0.82)	12.2 (11.1; 14.2)	587 (365; 800)	-1.42 (-2.18; -0.70)	208 (100)
Senegal	88	870	95	8.9 (6.3; 9.8)	362 (125; 485)	-2.10 (-2.87; -1.15)	12.5 (11.1; 14.5)	511 (342; 797)	-1.68 (-2.56; -0.51)	81 (92.1)
Kenya	5913	710	94	8.2 (6.4; 9.7)	344 (188; 588)	-1.85 (-2.85; -0.85)	11.8 (10.8; 13.6)	667 (427; 935)	-1.73 (-2.58; -0.89)	4580 (77.5)
Rwanda	1244	370	91	7.8 (6.3; 9.2)	441 (289; 783)	-2.04 (-3.14; -1.09)	12.1 (10.9; 13.7)	692 (493; 945)	-1.90 (-2.77; -1.00)	906 (72.8)

Tanzania	1521	510	85	8.8 (7.2; 9.9)	275 (130; 432)	-2.06 (-3.02; -1.11)	11.7 (10.7; 13.1)	584 (320; 867)	-2.13 (-2.90; -1.31)	1207 (79.4)
Burkina Faso	122	460	81	7.4 (5.6; 8.8)	358 (230; 580)	-1.70 (-2.35; -1.00)	12.4 (11.3; 14.5)	729 (538; 963)	-1.04 (-1.92; -0.15)	119 (97.5)
Burundi	66	170	65	8.9 (7.6; 10.6)	418 (343; 516)	-1.80 (-2.11; -1.08)	11.7 (10.7; 12.6)	875 (654; 1004)	-1.45 (-2.41; -0.91)	49 (74.2)
Zimbabwe	952	380	63	8.4 (7.1; 9.4)	251 (137; 378)	-2.02 (-2.88; -1.21)	12.3 (10.9; 13.8)	677 (395; 945)	-1.84 (-2.55; -1.11)	915 (96.11)
Malawi	1653	320	57	7.9 (5.5; 9.5)	254 (156; 380)	-2.46 (-3.42; -1.58)	12.1 (10.8; 13.8)	677 (437; 1006)	-2.07 (-2.81; -1.33)	1387 (83.9)
Mozambique	1523	400	41	8.6 (7.2; 9.6)	359 (198; 626)	-2.00 (-2.80; -1.22)	11.4 (10.6; 12.7)	598 (339; 911)	-2.04 (-2.83; -1.17)	1315 (86.3)
Ethiopia	1761	220	38	7.8 (6.1; 9.0)	329 (173; 595)	-1.84; (-2.70; -0.80)	11.8 (10.8; 13.1)	641 (427; 888)	-1.93 (-2.76; -1.10)	1455 (82.6)
Guinea	75	310	35	8.2 (7.1; 9.2)	205 (108; 307)	-1.93 (-2.72; -0.96)	11.8 (10.8; 12.7)	560 (344; 793)	-1.57 (-2.34; -0.86)	65 (86.7)
DRC	402	250	19	7.7 (5.9; 9.2)	317 (157; 554)	-1.76 (-2.86; -0.87)	12.7 (11.1; 14.3)	722 (450; 1049)	-1.21 (-0.20; -0.37)	364 (90.6)

ART – antiretroviral therapy; CAR – Central African Republic; DRC – Democratic Republic of Congo; Gross National Income in US Dollars per capita;

[#] As estimated by the World Bank in US Dollars for the year 2007

Supplementary Table 4: Individual country mortality hazard ratios

Country	N	GNI#	Health Spending#	uHR	aHR*
**Upper Middle Income**					
South Africa	5445	5940	837	Reference	Reference
Botswana	540	5620	580	0.43 (0.13; 1.38)	0.54 (0.17; 1.77)
**Lower Middle Income**					
Swaziland	555	3190	388	2.79 (1.48; 5.27)	2.63 (1.31; 5.27)
Lesotho	793	1310	155	3.63 (2.17; 6.09)	3.02 (1.72; 5.30)
**Low Income**					
Cote d'Ivoire	635	970	161	4.11 (2.44; 6.97)	4.04 (2.30; 7.09)
Uganda	2313	370	155	3.19 (2.21; 4.62)	2.61 (1.70; 4.01)
Ghana	148	800	137	2.10 (0.51; 8.64)	2.02 (0.49; 8.40)
Zambia	4224	880	113	1.89 (1.29; 2.75)	1.54 (1.03; 2.31)
Mali	208	540	96	5.08 (2.49; 10.38)	4.06 (1.95; 8.45)
Senegal	88	870	95	7.66 (3.27; 17.93)	7.31 (3.08; 17.34)
Kenya	5913	710	94	2.60 (1.85; 3.66)	2.78 (1.90; 4.07)
Rwanda	1244	370	91	0.94 (0.46; 1.91)	0.68 (0.27; 1.74)
Tanzania	1521	510	85	5.25 (3.52; 7.84)	5.44 (3.51; 8.44)
Burkina Faso	122	460	81	<0.0000	<0.0000
Burundi	66	170	65	NA	NA
Zimbabwe	952	380	63	2.74 (1.62; 4.64)	2.11 (1.21; 3.70)
Malawi	1653	320	57	3.55 (2.35; 5.36)	3.15 (2.00; 4.98)
Mozambique	1523	400	41	3.77 (2.40; 5.94)	3.66 (2.24; 6.00)
Ethiopia	1761	220	38	1.98 (1.19; 3.29)	1.52 (0.84; 2.74)
Guinea	75	310	35	4.58 (1.11; 18.90)	2.29 (0.31; 16.66)
DRC	402	250	19	5.46 (3.20; 9.32)	4.88 (2.71; 8.78)

GNI – Gross National Income; CAR – Central Afrian Republic; DRC – Democratic Republic of Congo; aHR – adjusted hazard ratio; uHR – unadjusted hazard ratio;

As estimated by the World Bank in US Dollars for the year 2007

* Restricted to those ever on ART and adjusted for sex, birth cohort, first visit – age, -CD4 count, -WAZ, - HAZ
