# Supplementary figures and images for: Inequality in outcomes for adolescents living with perinatally acquired HIV in sub‐Saharan Africa: a Collaborative Initiative for Paediatric HIV Education and Research (CIPHER) Cohort Collaboration analysis
Source: J Int AIDS Soc. 2018 Feb 27;21(Suppl Suppl 1):e25044. doi: 10.1002/jia2.25044 (PMC5978669; doi:10.1002/jia2.25044)

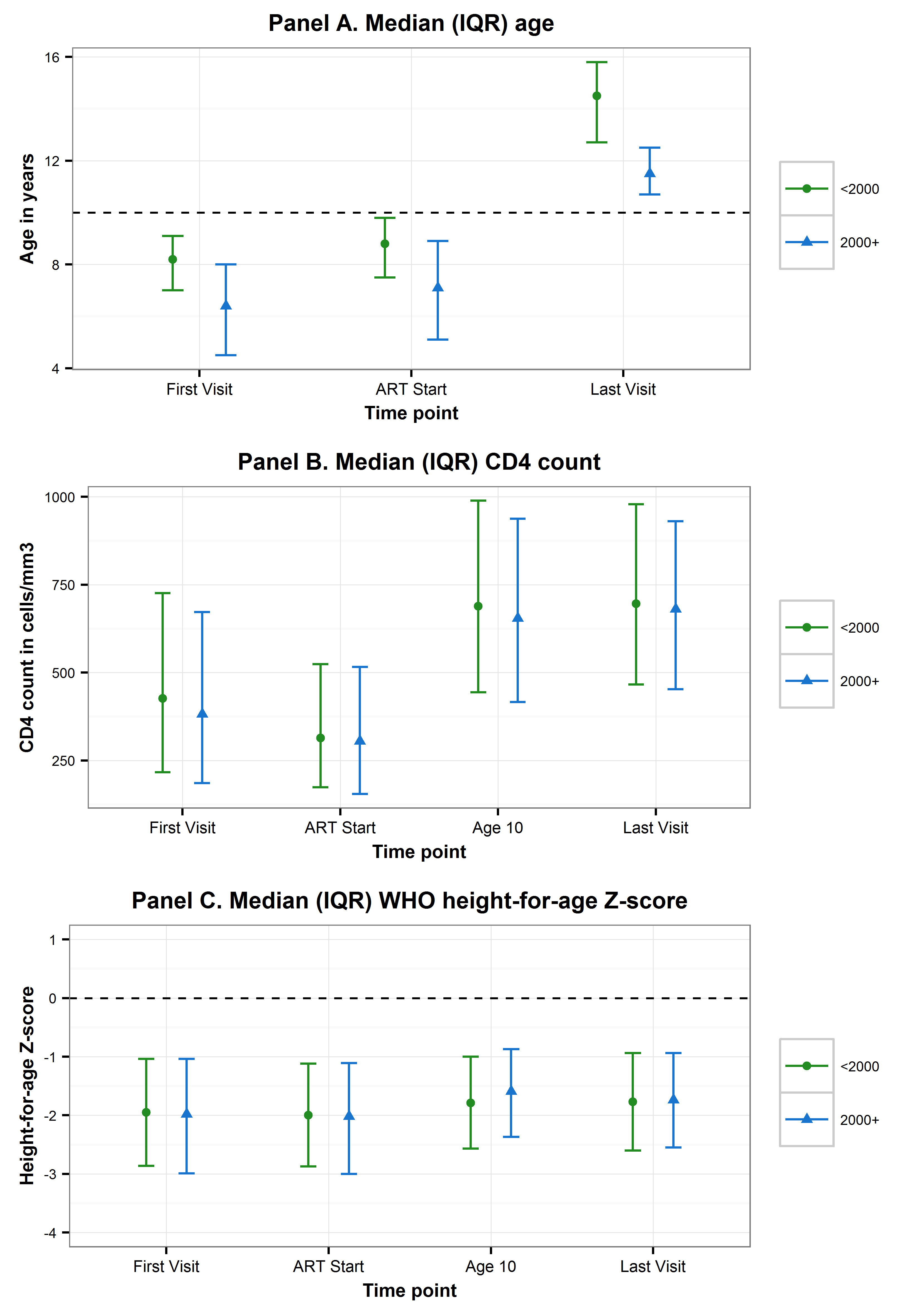

Supplement: Supplementary file 2 — Figure S1 Graphic comparison by birth cohort of characteristics at first visit, ART start, age 10 years and last visit of adolescents living with perinatally acquired HIV. [file JIA2-21-e25044-s002.tiff]
